# Implicit Temporal Expectation Attenuates Auditory Attentional Blink

**DOI:** 10.1371/journal.pone.0036031

**Published:** 2012-04-27

**Authors:** Dawei Shen, Claude Alain

**Affiliations:** 1 Rotman Research Institute, Baycrest Centre for Geriatric Care, Toronto, Ontario, Canada; 2 Department of Psychology, University of Toronto, Toronto, Ontario, Canada; 3 Institute of Medical Sciences, University of Toronto, Toronto, Ontario, Canada; National University of Singapore, Singapore

## Abstract

Attentional blink (AB) describes a phenomenon whereby correct identification of a first target impairs the processing of a second target (i.e., probe) nearby in time. Evidence suggests that explicit attention orienting in the time domain can attenuate the AB. Here, we used scalp-recorded, event-related potentials to examine whether auditory AB is also sensitive to implicit temporal attention orienting. Expectations were set up implicitly by varying the probability (i.e., 80% or 20%) that the probe would occur at the +2 or +8 position following target presentation. Participants showed a significant AB, which was reduced with the increased probe probability at the +2 position. The probe probability effect was paralleled by an increase in P3b amplitude elicited by the probe. The results suggest that implicit temporal attention orienting can facilitate short-term consolidation of the probe and attenuate auditory AB.

## Introduction

Attentional blink (AB) refers to the phenomenon whereby correct identification of a first target (target or T1) causes a processing deficit for a second target (probe or T2) when the two are presented in close succession amongst distracters in a rapid, serial, auditory/visual presentation. This ‘attentional blink’ persists for several hundred milliseconds and it provides important information about attentional allocation in the temporal domain [1]–[7].

During the last decade there has been a growing interest in identifying the optimal conditions to overcome the processing limitation during AB. Behavioral studies have shown that visual AB can be reduced by guiding attention toward the probe via either visual cues [8]–[10] or task instructions [11]. There is also evidence from scalp recording of event-related potentials (ERPs) that auditory AB can be modulated by instructing participants to focus their attention to a specific time interval within a sequence of stimuli [12]. Together, these studies indicate that there is some degree of flexibility in the allocation of processing resources, despite the existence of processing bottlenecks [13], and that attention can be directed toward a probe feature [11] or temporal position [9], [12], thereby facilitating its processing.

To date, the effect of attention orienting on AB has been demonstrated using explicit attentional manipulation. However, it remains to be determined whether or not implicit attention orienting can attenuate AB. Contrary to explicit expectation, which is a top-down process, implicit expectation arises from bottom-up processing and requires the ability to optimally distribute cognitive resources. Evidence from prior research suggests that attention can be allocated toward a specific point in time using implicit manipulation. For instance, Doherty et al. [14] set up spatial or temporal expectation implicitly using a moving visual stimulus. They found that temporal expectation set up implicitly by the moving stimulus reduced reaction time, which coincided with an earlier and larger P3b wave, a positive deflection from the ERPs that is largest at parietal scalp sites between 300 and 600 ms after a target stimulus. Rimmele et al. [15] used a moving auditory stimulus and revealed that temporal expectation speeded reaction time and increased sensitivity (d′) as well as P3b amplitudes. Muller-Gethmann et al. [16] employed a foreperiod paradigm (i.e., manipulating the time interval between a warning signal and the target, and participants were not explicitly told about this manipulation). They found that the temporal expectation affected reaction time and the P3b wave. The P3b amplitude was smallest for the foreperiods with optimal preparation while its latency increased with the increasing of the foreperiod. In a different study, Los and Heslenfeld [17] used sequential effects of foreperiod and revealed an effect of implicit temporal expectation on reaction time and the amplitude of the contingent negative variation (CNV). Together, these studies provide converging evidence suggesting that implicit manipulation can successfully bias attention toward a specific time thereby facilitating target processing.

In the present study, we examined whether implicit temporal expectation would also attenuate AB. Temporal expectation was set up by varying the probability (i.e., 80% or 20%) that the probe would occur at the +2 or +8 position following the target in a stream of sounds. Such probability manipulation has been successful in visual search studies where visual attention has been biased towards a particular location by varying the target's spatial probability [18]–[20]. For instance, in the study of Shaw and Shaw [20], a letter could be presented at one of eight clockwise positions. Different positions had different probabilities (25%, 10% or 5%). Participants were more accurate at identifying a target letter (E, T, or V) when it occurred at the highest probability location. They proposed a capacity allocation model to explain the spatial probability effect. That is, participants allocated more resources to the high probability location thereby improving accuracy. In the present study, we hypothesized that increasing probe probability at a particular temporal position would bias attention and improve probe detection.

In addition to behavioral measurements, we also recorded ERPs to further reveal how probe probabilities affected processing resource allocation during auditory AB. Prior ERP studies of visual and auditory AB have shown a suppression of the P3b wave elicited by the probe [21]–[26]. The P3b wave is thought to index the updating of working memory [27] and/or represent the transfer of information to consciousness [28]. Its suppression during the AB interval may indicate a deficit in consolidation in which the probe did not reach the capacity-limited short-term consolidation stage [26]. We hypothesized that 1) reduced auditory AB would be observed when attention was oriented towards the probe position and 2) that reduced AB would coincide with an increase in P3b amplitude which may be explained by increased processing resources being allocated to that position.

## Results

### Behavioral Results

#### Target Detection

Table 1 shows the group mean target detection accuracy. The effect of probe position on target accuracy was significant, *F* (1, 15) = 5.53, *p*<.05. Pairwise comparisons revealed that participants were more accurate at target detection when the probe was presented at the +8 position than at the +2 position or when no probe was presented (*p*<.05). There was no difference in target detection between the +2 and no probe condition. All other effects were not significant.

**Table 1:** Target detection accuracy (mean and standard error) as a function of probe probability, probe presence and probe position when the target was present.

Probe Probability	Probe Presence		
	Yes		No
	+2	+8	
80% at +2 & 20% at +8	.91 (.02)	.93 (.02)	.90 (.02)
20% at +2 & 80% at +8	.90 (.02)	.92 (.02)	.90 (.02)

#### Probe Detection

**Figure 1:**
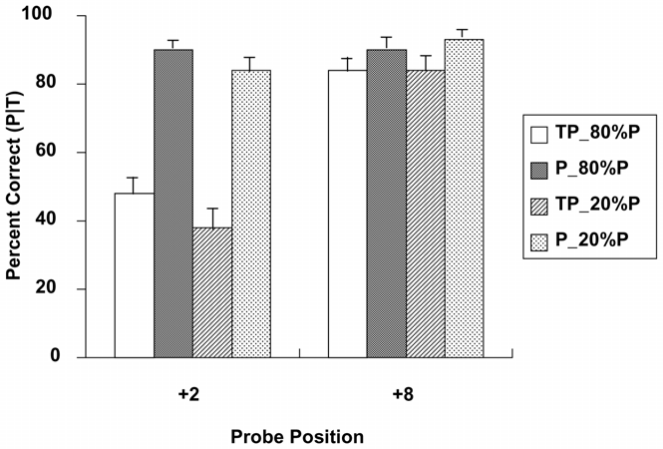
Probe detection accuracy as a function of the target presence, of the probe position, and of probe probability. Error bars represent +1 standard errors.

Figure 1 shows the group mean accuracy for probe detection in the low and high probe probability conditions at the +2 and +8 positions.

We conducted a 2 (probe probability)×2 (target presence)×2 (probe position) within-subjects ANOVA, which was performed on the conditional probability of accurate probe detection given a correct target detection response. The ANOVA revealed main effects of probe probability, *F*(1, 15) = 9.82, *p*<.01, target presence, *F*(1, 15) = 73.35, *p*<.001, and probe position, *F*(1, 15) = 95.30, *p*<.001. In general, the probe detection was better when the target was absent, when the probe was presented at the +8 position, and when the probe probability was 80%.

There were significant two-way interactions between probe probability and target presence, *F* (1, 15) = 4.78, *p*<.01, between target presence and probe position, *F*(1, 15) = 75.64, *p*<.001, and between probe probability and probe position (F(1, 15) = 6.46, *p*<.05. The three-way interaction between probe probability, target presence, and probe position was not significant, *F<*1.

The main effect of target presence and the significant interaction between target presence and probe position on probe processing accuracy are taken as evidence that a significant auditory AB has occurred [29]. Moreover, the significant two-way interaction between probe probability and target presence indicated that the AB was affected by the probe probability. That is, the AB was reduced when the probe probability was higher.

Separate ANOVAs were conducted for the +2 and +8 position to further assess the effects of probe probability and target presence on probe detection within and outside the AB window. When the probe was at the +2 position (i.e., within AB window), accuracy was higher 1) when the probe probability was 80% than when it was 20% (*F*(1, 15) = 9.18, *p*<.01), and 2) when the target was absent versus when it was present, *F*(1, 15) = 88.48, *p*<.001. The interaction between probe probability and target presence was not significance, *F<*1. When the probe was at the +8 position (i.e., outside of AB window), accuracy was similar for the 20% or 80% probe probability (*F<*1), and was higher when the target was absent than when it was present, *F*(1, 15) = 11.08, *p*<.01. The interaction between the probe probability and target presence was not significant, *F*(1, 15) = 1.90, *p* = .19.

### Electrophysiological Data

**Figure 2:**
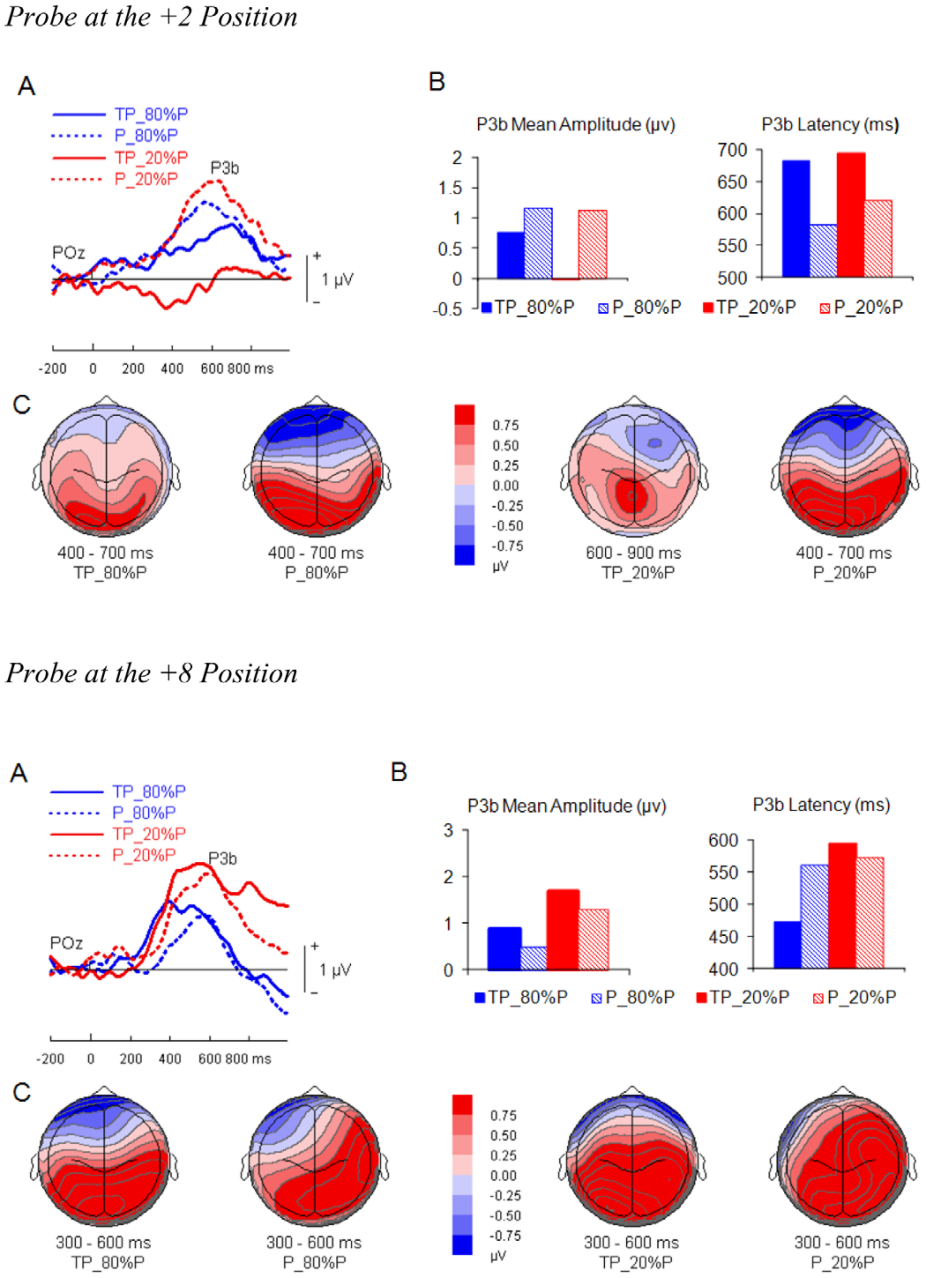
ERPs elicited by the probe at the +2 and the +8 positions. Group mean difference waves of probe-locked ERPs elicited by target-probe sequences minus target only sequences as a function of probe probability. Similarly, difference waves of probe-locked ERPs elicited by probe only sequences minus the sequences with neither the target nor the probe as a function of probe probability. A: traces from the parietal electrode POz. B: mean amplitude and latency from parietal electrodes (i.e., P3, Pz, P4, PO3, POz, and PO4). C: the bottom panel shows the topographic P3b amplitude distribution from the corresponding experimental conditions during peak latency. TP_80%P: both target and probe were present and 80% probe at the designated temporal position; P_80%P: only probe was present and 80% probe at the designated temporal position; TP_20%P: both target and probe were present and 20% probe at the designated temporal position; P_20%P: only probe was present and 20% probe at the designated temporal position.

In our AB paradigm, both target and probe were task-relevant and therefore elicited a P3b wave [28]. However, when the target and the probe were close in time, the P3b waves elicited by the target and the probe overlapped and the P3b specific to the probe could not easily be quantified. To circumvent this problem, the ERPs elicited by target only were subtracted from ERPs elicited by both target and probe [23]. This subtraction procedure revealed a positive slow wave (i.e., P3b) that was maximal over the parietal and parieto-occipital scalp region (Figure 2). In addition to this difference wave, we also subtracted ERPs elicited by neither target nor probe from ERPs elicited by probe only. Both difference waves revealed P3b responses specific to the probe without contamination of auditory steady state responses elicited during serial rapid auditory presentation.

A 2 (probe probability)×2 (target presence)×2 (probe position) within-subjects ANOVA on the P3b mean amplitude of the probe was conducted. The P3b mean amplitude was slightly larger when the probe was presented at the +8 position than when it was at the +2 position, *F*(1, 15) = 3.22, *p* = .09. There were significant interactions between probe probability and probe position, *F*(1, 15) = 7.15, *p*<.05, and between target presence and probe position, *F*(1, 15) = 11.16, *p*<.01. The three-way interaction between probability, target presence, and probe position approached significance, *F*(1, 15) = 3.29, *p* = .09.

Separate ANOVAs were conducted for the +2 and +8 positions to assess the effects of probe probability and target presence on P3b amplitude within and outside the AB window. When the probe was at the +2 position, the P3b mean amplitude was larger when the probe was presented alone than when it followed the target, *F*(1, 15) = 6.44, *p*<.05. The main effect of probability approached significance, *F*(1, 15) = 3.30, *p* = .09. There was a significant interaction between probe probability and target presence, *F*(1, 15) = 4.61, *p*<.05. The P3b amplitude was larger for probe probability of 80% versus that of 20% when the probe followed the target *p*<.05. The effect of probe probability on the difference waves for probe only was not significant. When the probe was at the +8 position, the P3b mean amplitude was larger when the probe was 20% than when it was 80%, *F*(1, 15) = 6.52, *p*<.05. No other effects reached significance, *p*>.10 for all cases.

The analysis of P3b latency revealed significantly shorter P3b latency when the probe was 80% than when it was 20%, *F*(1, 15) = 7.51, *p*<.001, and when the probe was presented at the +8 position than when it was at the +2 position, *F*(1, 15) = 25.85, *p*<.001. The P3b latency was also slightly shorter when the target was absent than when it was present, *F*(1, 15) = 3.49, *p* = .08. There were significant interactions between target presence and probe position, *F*(1, 15) = 8.98, *p*<.01. The three-way interaction between probability, target presence, and probe position approached significance, *F*(1, 15) = 3.33, *p* = .09. No other effects reached significance, *p*>.10 for all cases.

Separate ANOVAs were conducted for the +2 and +8 position to assess the effects of probe probability and target presence on P3b latency within and outside the AB window. When the probe was at the +2 position, the P3b latency was longer when the probe followed the target than when the probe was alone, *F*(1, 15) = 9.25, *p*<.01. The main effect of probability was not significant (*F*(1, 15) = 2.33, *p* = .15), nor was the interaction between probability and target presence. For the +8 position, the analysis revealed longer P3b latency when the probe probability was 20% than when it was 80%. There was a significant interaction between probe probability and target presence, *F*(1, 15) = 7.56, *p*<.05. In trials where the target was present, the latency was longer when the probe probability was 20% than when it was 80%. However, when the target was absent, there was no such a difference, *F*<1.

## Discussion

There is increasing evidence that guiding visual or auditory attention toward a probe using explicit cues or instructions can attenuate the AB [8]–[12]. Here, by varying probe probability, we provide new evidence that attention can also be biased implicitly toward a specific temporal position thereby easing probe detection within the AB window. This attenuation of the auditory AB was paralleled by increased P3b amplitude elicited by the probe. Together, the behavioral and electrophysiological data provide converging evidence that the allocation of processing resources can be induced implicitly, and that temporal attention orienting can partly overcome the processing limitation reflected in the AB.

There are several mechanisms by which temporal attention orienting may reduce the magnitude of the AB. For instance, temporal expectation generated by the increasing probe probability at a specific temporal position within the auditory sequence may reduce the threshold for probe detection and recognition. This would be analogous to the effect of selective attention on task-relevant stimuli designated by their location [30], [31] or frequency [32], [33]. That is, increasing probe probability at a specific temporal position allows participants to anticipate when the probe would occur thereby easing its detection among the stream of distractors.

The P3b wave recorded during visual and auditory AB has been proposed as an index of processes engaged during the short-term consolidation stage [25], [26]. Because short-term consolidation is capacity-limited, the target and the probe could not be processed at the same time. The reduced P3b wave elicited by the probe during the AB can perhaps be explained by the possibility that the processing of some probes does not reach the consolidation stage because they are overwritten or passively decay during the time the participant is waiting for the completion of target processing. Accordingly, this waiting results in the delay of P3b waves [12], [21], [25], [34]. From this perspective, the increase in accuracy and P3b amplitude during the AB would indicate that the probe reached the capacity-limited short-term consolidation more often when attention was implicitly allocated at the expected time as predicted by the probe probability.

The effects of implicit temporal orienting share similarities with those of explicit manipulation observed in a prior auditory AB study [12]. In the present study, the P3b evoked by the probe at the +8 position peaked earlier when attention was implicitly oriented at that temporal position. Similarly, our prior work showed that explicit task instruction also yielded earlier P3b at the +8 as well as the +4 position [12]. One mechanism by which temporal orienting could promote short-term consolidation is by substitution [35], the process whereby old items are replaced by new items in working memory. High expectancy of the probe's presence might result in a quick removal of the target and thus the probe's short-term consolidation would be sped up. Alternatively, the short-term consolidation process may remain “idle" after the target processing ended and this would result in a quick initiating of the probe encoding. In both studies, the effect of implicit and explicit manipulation on the P3b latency elicited at the +1 or +2 position was difficult to assess because there was no reliable P3b wave when attention was not allocated at the +1 or +2 position. Further research using a presentation rate manipulation [25] may help determine whether implicit and/or explicit manipulation would also modulate the P3b latency at the +1 or +2 position.

Researchers have shown that P3b amplitude is affected by the allocation of attention when equivocation (amount of information loss) is high [36]. During the AB, there is a large amount of information loss with respect to probe detection. Thus, the allocation of attention would be an important factor in affecting the P3b amplitude during the AB. Another well-known factor is the ‘oddball’ effect, an inverse relation between stimulus probability and P3b amplitude [27], [36]–[40]. Previous studies have revealed that the ‘oddball’ effect is attenuated or eliminated when target items are difficult to process [36], [41]–[44]. Thus, during the AB, the allocation of attention would be the main factor affecting the P3b amplitude.

When the probe was at the +8 position (i.e., outside of the AB window), the P3b amplitude was larger for probe probabilities of 20% than those of 80%. This result was consistent with the expectation that the ‘oddball’ effect would be a main factor affecting the P3b amplitude of the probe but that the allocation of attention would have little effect on the P3b amplitude when the probe was outside the AB window and therefore more easily processed.

In brief, probe detection during the auditory AB was modulated by varying the probe probability without making the participants explicitly aware of this manipulation. The changes in probe detection were paralleled by changes in P3b amplitude consistent with the short-term consolidation hypothesis. The behavioral and electrophysiological data provide converging evidence that auditory AB can be mediated by implicitly guiding attention to the probe temporal position thereby easing its consolidation in short-term memory. In addition to AB studies, previous studies on temporal attention [45], [46] have also revealed larger and earlier P3b wave when temporal attention was cued to the target than it was not. Together, these findings suggest that temporal orienting can enhance cognitive performance in general.

## Materials and Methods

### Participants

Sixteen young adults (age: 18 to 30 years old, 9 females) participated in this study. They had normal hearing as measured by pure tone thresholds (i.e., hearing thresholds less than or equal to 20 dB for octave pure tone frequencies ranging from 250 to 8000 Hz). Ethical approval for this experiment was obtained by the Baycrest Research Ethics Board and the participants provided their written informed consent using a Baycrest Research Ethics Board approved consent form.

### Stimuli

Twenty-one pure tones were used as distractors. The frequencies of these tones ranged from 529 to 1330 Hz. The specific frequencies were 529, 554, 580, 607, 636, 666, 697, 730, 764, 800, 838, 877, 918, 961, 1006, 1056, 1106, 1158, 1213, 1270, and 1330 Hz. The target was composed of six 5-ms pulses and its frequency could be any of the 21 frequencies of the distractors. The probe was a tone glide, i.e., a sound that increased continuously in frequency from 636 to 1006 Hz within its duration. All sounds were synthesized using Adobe Audition 1.5 at a sampling rate of 44100 Hz and were 30 ms in duration, including 2- ms linear onset/offset amplitude ramp to eliminate onset/offset clicks. Stimulus presentation was controlled using a Dell Precision T3400 computer running the Neurobehavioral Systems Presentation 13.0. Sounds were presented at a comfortable intensity of about 75 dB SPL through Etymotic ER3A insert earphones.

### Procedure

Each trial consisted of a sequence of 16 sounds. The stimulus onset asynchrony (SOA) between two successive sounds was 120 ms. The target was presented at the fifth temporal position and the probe could be presented at the second (i.e., +2) or the eighth temporal position (i.e., +8) following the target. The other sounds were considered distractors. A distractor sound would be present at the same position when the target or the probe were not present. The probability (i.e., 20% or 80%) to have the probe at the +2 and +8 position was manipulated in separate block of trials. These probabilities were chosen to bias participants' attention toward a particular temporal position within the sequence (i.e., +2 or +8 position).

In each probability condition there were six different trial types: (i) target and probe were both presented, and the probe was presented at +2 positions; (ii) probe was presented alone at +2 position; (iii) target and probe were both presented, and the probe was presented at +8 position; (iv) probe was presented alone at +8 position; (v) target was presented alone; and (vi) neither target nor probe were presented. In the 80% probe at +2 condition (i.e., 20% probe at the +8 position), there were 80 trials for each of type (iii), (iv), (v), and (vi), but 320 trials for each of type (i) and (ii). In the 80% probe at +8 condition (i.e., 20% probe at the +2 position), there were 80 trials for each of type (i), (ii), (v) and (vi), but 320 trials for each of type (iii) and (iv). At the end of each trial (i.e., sequence of 16 tones), participants made separate judgments for the presence of the target and the probe. The first question asked was, ‘Was the target presented, yes [press 1] or no [press 2]?’ the second question asked was, ‘Was the probe presented, yes [press 1] or no [press 2]?’ Note that participants were given no instructions concerning temporal probe positions or probe probabilities. Each participant met a criterion of 60% correct judgment on both the target and the probe in the practice blocks before beginning the study. Each participant completed the two probability conditions in two separate sessions one week apart. Condition order was counter-balanced. In each condition, there was one block of practice trials (24 trials) and four blocks of experimental trials (240 trials for each block), and participants could take a short break after each block.

### Electrophysiological recording and analysis

Neuroelectric brain activity was digitized continuously with a bandpass of 0.16–100 Hz and a sampling rate of 512 Hz using a BioSemi Active Two System (BioSemi V. O. F., Amsterdam, Netherlands). The electroencephalogram was recorded from 64 scalp electrodes based on the 10/20 system in a Biosemi electrode cap, with a Common Mode Sense (CMS) active electrode and Driven Right Leg (DRN) passive electrode serving as ground. Ten additional electrodes placed below the hair line (both mastoid, both pre-auricular points, outer canthus of each eye, inferior orbit of each eye, two facial electrodes) to monitor eye movements and to cover the whole scalp evenly. The latter is important because we used an average reference (i.e., the average of all scalp EEG channels as the reference for each EEG channel) for ERP analyses. All off-line analyses were performed using Brain Electrical Source Analysis software (BESA, version 5.2.4; MEGIS GmbH, Gräfelfing, Germany).

For each participant, a set of ocular movements was obtained prior to and after the experiment [47]. From this, average lateral and vertical eye movements were calculated as well as eye-blinks. A principal component analysis of these averaged recordings provided a set of components that best explained the eye movements. The scalp projections of these components were then subtracted from the experimental ERPs to minimize ocular contamination such as blinks, saccades and lateral eye movements for each individual average.

The epoch included 200 ms of pre-stimulus activity and 1000 ms of post-stimulus activity to highlight the time course of neural activity following the probe (i.e., the ERPs were time-locked to the onset the probe). ERPs were averaged separately for each target present or probe present condition (target and probe, target only, probe only, neither), probe position (2^nd^ or 8^th^ position following the target), probe probability (20% or 80%), participant, and electrode site. Before measurement, the ERPs were digitally filtered to attenuate frequencies above 20 Hz (24 dB/octave attenuation, symmetrical, zero phase).

### Data Analysis

We used a 2 (probe probability)×2 (target presence)×2 (probe presence)×2 (probe position) within subject design.

First, we examined the effects of probe probability, probe presence, and probe position on target detection. Then, to assess the effects of temporal orienting on auditory AB, we tested for the effects of probe probability on probe detection at the +2 and +8 positions when the probe occurred frequently (i.e., 80%) versus occasionally (i.e., 20%). The probe at the +2 position was 240 ms following the target onset, which was within the AB window and was the AB condition. In contrast, the probe at the +8 position occurred 960 ms following the target onset, which was outside the AB window, and was a control condition in the present study. The analyses were performed on the conditional probability of accurate probe detection given a correct target detection response.

Following previous AB studies [25], [34], we examined the effects of probe probability on the P3b mean amplitude measured during the 300–900 ms interval following the probe onset at the parietal sites (i.e., P3, Pz, P4, PO3, POz, and PO4). This electrode array was chosen because it provides a reliable estimate of the P3b, which is typically largest at parietal sites. Electrode was treated as a variable when being entered into ANOVA. However, we did not compare the effects between different electrodes since it was not the focus of the present study. The P3b peak latency was defined as the maximum positivity between 300 and 900 ms after probe onset, also at parietal sites. When appropriate, the degrees of freedom were adjusted with the Greenhouse-Geisser epsilon (ε) to correct for inhomogeneity of variance and all reported probability estimates are based on the reduced degrees of freedom, although the original degrees of freedom are reported.
